# Establishment and characterization of two human breast carcinoma cell lines by spontaneous immortalization: Discordance between Estrogen, Progesterone and HER2/neu receptors of breast carcinoma tissues with derived cell lines

**DOI:** 10.1186/1475-2867-12-43

**Published:** 2012-10-29

**Authors:** Behnam Kamalidehghan, Massoud Houshmand, Fereydoun Kamalidehghan, Narges Jafarzadeh, Shahram Azari, Sharifah Noor Akmal, Rozita Rosli

**Affiliations:** 1 Genetic Medicine Research Centre, Faculty of Medicine and Health Sciences, Universiti Putra Malaysia UPM Serdang, Selangor, 43400, Malaysia; 2 Department of Pharmacy, Faculty of Medicine, University of Malaya, 50603 Kuala Lumpur, Malaysia; 3 Department of Medical Genetics, National Institute of Genetic Engineering and Biotechnology (NIGEB), Tehran, Iran; 4 Genetics Department, Special Medical Center, Tehran, Iran; 5 Pazhohan Teb Co Ltd, Karaj, Tehran, Iran; 6 National Cell Bank of Iran, Pasteur Institute of Iran, Tehran, Iran; 7 Department of Molecular Medicine, School of Advanced Technology of Medical Sciences, Golestan University of Medical Science, Gorgan, Iran; 8 Department of Pathology, Faculty of Medicine, Universiti Kebangsaan Malaysia, Jalan Yaacob Latif Cheras, Kuala Lumpur, 56000, Malaysia; 9 UPM-MAKNA Cancer Research Laboratory, Institute of Bioscience, Universiti Putra Malaysia UPM Serdang, Selangor, 43400, Malaysia

**Keywords:** Malaysian breast cell lines, Estrogen (ER), Progesterone (PR) and HER2/neu, Clonogenic assay, Invasive ductal breast carcinoma tissues, DNA profiling

## Abstract

**Background:**

Breast cancer is one of the most common cancers among women throughout the world. Therefore, established cell lines are widely used as *in vitro* experimental models in cancer research.

**Methods:**

Two continuous human breast cell lines, designated MBC1 and MBC2, were successfully established and characterized from invasive ductal breast carcinoma tissues of Malaysian patients. MBC1 and MBC2 have been characterized in terms of morphology analysis, population doubling time, clonogenic formation, wound healing assay, invasion assay, cell cycle, DNA profiling, fluorescence immunocytochemistry, Western blotting and karyotyping.

**Results:**

MBC1 and MBC2 exhibited adherent monolayer epithelial morphology at a passage number of 150. Receptor status of MBC1 and MBC2 show (ER^+^, PR^+^, HER2^+^) and (ER^+^, PR^-^, HER2^+^), respectively. These results are in discordance with histopathological studies of the tumoral tissues, which were triple negative and (ER^-^, PR^-^, HER2^+^) for MBC1 and MBC2, respectively. Both cell lines were capable of growing in soft agar culture, which suggests their metastatic potential. The MBC1 and MBC2 metaphase spreads showed an abnormal karyotype, including hyperdiploidy and complex rearrangements with modes of 52–58 chromosomes per cell.

**Conclusions:**

Loss or gain in secondary properties, deregulation and specific genetic changes possibly conferred receptor changes during the culturing of tumoral cells. Thus, we hypothesize that, among heterogenous tumoral cells, only a small minority of ER^+^/PR^+^/HER2^+^ and ER^+^/PR^-^/HER2^+^ cells with lower energy metabolism might survive and adjust easily to *in vitro* conditions. These cell lines will pave the way for new perspectives in genetic and biological investigations, drug resistance and chemotherapy studies, and would serve as prototype models in Malaysian breast carcinogenesis investigations.

## Introduction

Breast cancer is the most common cancer among women throughout the world. The number of newly-diagnosed cases in 2008 was approximately 1.38 million, and the number of deaths was more than 460,000 worldwide [1].

Breast cancer is the most common cancer among Malaysian women. The peak age for cancer cases among the three major ethnic groups, namely Malay, Chinese and Indian was between 40 to 49 years [2]. Malay women presented larger tumours, in which 50% to 60% were at the later stages (3 and 4), compared to other ethnic groups [3].

Though advances in breast cancer detection and treatment have contributed to improving the rate of survival, mortality rates remains significantly high. The establishment of breast cancer cell lines is an important tool to understand biological processes involved in this disease, as well as the identification of potential therapeutic targets [4].

Cell lines are used to determine key initiation and progression factors in order to better understand a disease. They are also used to predict the efficacy of novel therapeutic compounds. However, the cell lines currently used for breast cancer are mostly derived from Caucasians or African-Americans. Though breast cancer cell lines are easy to handle and replaced from frozen stocks, they are prone to genotypic and phenotypic drifts during consecutive cultures [5,6].

Most *in vitro* studies using a few well-characterized breast cancer cell lines such as MCF-7, MDA-MB-231, T-47D and ZR-75-30 have been established for over 30 years. These cell lines were derived from tumor metastases especially aspirate or pleural effusions but not from primary breast tumors [7-9].

The main objective of this study was to establish and characterize two new immortalised Malaysian breast cancer cell lines, which are derived from two fresh primary invasive ductal breast carcinoma tissues.

## Materials and Methods

### Cancer cell isolation and cell culture

Ethics approval and patient informed consent including consent to participate in the study and consent to publish was obtained for the present study in accordance to the Universiti Kebangsaan Malaysia (UKM) Research and Medical Ethics Committee (Approval No. FF-166-2004).

Two invasive ductal breast carcinoma tissues, one from a Malay patient with triple negative (ER^-^, PR^-^, HER2^-^) tumor and another from a Chinese patient with (ER^-^, PR^-^, HER2^+^) tumor, were collected from Hospital Universiti Kebangsaan Malaysia (HUKM). After surgical removal, a portion of the primary breast tissues were immediately placed in FBS-free DMEM (Sigma-Aldrich, USA) and were minced and scraped to isolate cancer cells, in accordance to the manufacturer’s instructions of the Cancer Cell Isolation Kit (Panomics, USA). The cells were cultured in six-well culture plates supplemented with 10% FBS (Sigma-Aldrich, USA) and 3.7 g/L sodium bicarbonate (Sigma-Aldrich, USA) with a pH of 7.4 in a humidified incubator with 5% CO_2_ at 37°C (IR Sensor, Sanyo). Both cell lines were subcultured and then used to determine the characteristics of the established cell lines.

### Epithelial phenotype

The purity of the epithelial phenotype was confirmed by staining with a pan-cytokeratin antiserum (FITC conjugate; Dako, Denmark).

### Mycoplasma examination

Both MBC1 and MBC2 cell lines were cultured on coverslips in 6-well plates and incubated overnight until confluent. The coverslips were washed with PBS and fixed with a fixative; then stained with DAPI and kept in the dark place for 10 min. After that, the coverslips were washed with distilled water and dried to detect mycoplasma using a fluorescence microscope (Leica, Germany).

### Population doubling time (PDT)

A total of 2 × 10^5^ cells/ml of MBC1 and MBC2 cell lines at passage 30 were cultivated for a period of 3 days (24, 48 and 72 hrs). Cell numbers were determined every 24 hrs using a Neubauer improved haematocytometer (Sigma-Aldrich, USA) and cell numbers were counted in triplicate. Population doubling time was calculated using an online algorithm software provided at http://www.doubling-time.com*.*

### Clonogenic assay

A two-layer technique was used, with a base layer consisting of 1% agar (BST TechLab, Malaysia), and a second layer containing cells with 0.7% agar. Briefly, equal volumes of the base agar (melted in a microwave oven and cooled to 40°C in a water bath) containing 1% agar along with 2X DMEM containing 20% FCS were mixed to obtain 0.5% agar and 1X DMEM with 10% FCS. Subsequently, 1.5 ml of the mixed solution*s* was added to a petri dish (35 mm). The second layer, 3 ml of 2X DMEM (20% FCS) and 3 ml of 0.7% agar were added to a centrifuge falcon tube and mixed gently. The cells were trypsinised and counted to a ratio of 5 × 10^3^ cells per plate. After that, 1.5 ml was added to each petri dish (35 mm) that was covered with the earlier agar base. The plates were incubated for 14 days at 37°C and 5% CO_2_. Plates were then stained with 0.5 ml of 0.005% crystal violet for 2 hrs and colonies were counted using an inverted microscope Nikon Eclipse TS100 camera (Nikon DS-Fi1). The cloning efficiency (CE) was calculated as the number of colonies divided by the number of cells added to each plate.

### Wound healing assay (Migration assay)

The cell lines were seeded in 6-well plates until the cells reach confluence. Then, a straight scratch was made using a yellow plastic pipette tip. Next, the plates were rinsed twice with PBS to remove floating cells. The underside of the dish was marked to indicate the wounded area where the initial photos were taken, which allowed the imaging of both wound edges using the 10X objective at 6, 12, 18, 23 and 25 hrs. Subsequential images were periodically recorded with an inverted phase microscope Nikon Eclipse TS100 camera (Nikon DS-Fi1). The migration of cells across the wounds was expressed as the percentage of wound closure: Wound closure (%) = [(Initial area_t0_ - Final area_tf_)/Initial area_t0_] ×100.

### Matrigel invasion assay

The *in vitro* invasion assay was performed using Bio-Coat Matrigel invasion assay system (BD Biosciences, USA), according to the manufacturer’s instructions. Invaded cells were counted under a light microscope Nikon E600. The percentage of the invasion capability of the cell lines was calculated as follows: Invasion (%) =Number of cells attached to the lower side of the filter/total number of cells × 100.

### Genomic DNA preparation and PCR amplification

The genomic DNA from the cell lines was extracted using a Qiagen kit (QIAamp DNA Mini Kit, Germany), according to the manufacturer’s instructions. DNA yields were quantified by Nano-drop (Thermo Nanodrop 2000) and 1% agarose (Roche, Germany) gel electrophoresis to determine the integrity of extracted DNA. The details of the primer pairs for amplification of all 13 CODIS STR alleles and Amelogenin in this study was assessed according to the protocol previously described [10].

### Non-denaturing polyacrylamide gel electrophoresis

To achieve adequate resolution for the separation of the different STRs and Amelogenin alleles, large non-denaturing 10% polyacrylamide gels were used, as explained previously [10].

### Western blotting

After protein extraction from the cell lines using protein extraction buffer (Pierce, USA), 30 μg of proteins were separated by 12% SDS-PAGE (25 mA; 2 hrs). Proteins were transferred to PVDF (Pierce, USA) membranes using a Trans-Blot SD Semi-Dry Transfer Cell (Bio-Rad, USA) at 15 V, 95 mA, for 1 hour. The PVDF membrane was blocked using Blocker™ Casein (Pierce, USA) for 1 hour at room temperature and then washed twice using TBST. The membranes were then incubated at 4°C overnight with primary antibodies, estrogen mouse monoclonal antibody (1:1000; DAKO, Denmark), progesterone mouse monoclonal antibody (1:1000; DAKO, Denmark), HER2 rabbit polyclonal antibody (1:500; DAKO, Denmark) and β-actin mouse monoclonal antibody (1:10000; DAKO, Denmark). The membranes were then incubated for 1 hr at room temperature with goat anti-mouse and goat anti-rabbit secondary antibodies conjugated with alkaline phosphatase (i-DNA, USA) at a ratio of 1:1000 and then washed twice with TBST for 10 minutes three times on an orbital shaker. Then, blots were developed using the BCIP/NBT (Santa Cruz, USA) solution for a period of 5–30 minutes to detect the target protein band as a precipitated dark blue colour.

### Fluorescence immunocytochemistry of ER, PR and HER2

5×10^3^ cells were seeded on coverslips in 6-well plates and incubated at 37°C in a humidified CO_2_ incubator. The cells were washed with PBS and incubated with 700 μL of 4% (v/v) paraformaldehyde for 15 min at RT and then the cells were washed with PBS. Then, 700 μl of antigen retrieval buffer (100 mM Tris, 5% (w/v) urea, pH 9.5) was added to the wells for 10 min at 95°C and washed twice with PBS. Next, 700 μl of permeabilisation buffer (0.1% Triton X-100 in PBS) was added to the cells for 15 minutes at RT and then washed with PBS. The Blocking buffer (2% BSA) was added for 2 hrs and then washed with PBS. The primary antibodies (DAKO, Denmark) for estrogen, progesterone and HER2 was added at a ratio of 1:35, 1:50 and 1:250, respectively and left overnight, followed by washing with PBS. The goat anti-mouse FITC secondary antibody (i-DNA, USA) for ER and PR and the goat anti-rabbit FITC secondary antibody (i-DNA, USA) for HER2 were added for 1 hour at RT, incubated away from light and then washed twice with PBS. Finally, mounting buffer (Glycerol: PBS; 9:1) was used to mount coverslips on the slides to visualise ER, PR and HER2 expression under a fluorescent microscope (Leica, Germany).

### Determination of S-phase by the PI

Both cell lines were harvested and washed with 1X PBS; then, the cell pellets were fixed with pre-stored 90% ethanol at −20°C and were then incubated overnight at −20°C (0.5 ml). After incubation, cells were spun down at 800 rpm for 5 min. The supernatant was carefully discarded and the cells were resuspended by tapping until the pellet was fully dissolved. The cells were then washed in 1X PBS at 2000 rpm for 5 min. Again, supernatant was carefully discarded and the cells resuspended in 450 μl of pre-warmed 1X PBS and 25 μl of 10 mg/ml RNase at 37°C. After that, 50 μl of 1 mg/ml propidium iodide solution (PI) (Sigma-Aldrich, USA) was added and mixed well in an incubator for 30 min at 37°C. Finally, the stained samples were analysed using a FACS LSR FORTESSA flowcytometer which excites at 488 nm. The proliferation index (P.I.) was calculated by following formula P.I. = (S+G_2_M)/G_0_G_1_.

### Metaphase spread

The cell lines were seeded in DMEM medium to reach 70% confluent. Then, 50 μl of 10 μg/ml colcemid (GIBCO, USA) was added to the culture medium in a humidified incubator with 5% CO_2_ at 37Â°C for 2 hrs. Both media and trypsinised cells were centrifuged at 900 rpm for 5 min. The supernatant was decanted and the pellet was dispersed by tapping gently. Five ml of pre-warmed hypotonic solution (0.4% KCl and 0.4% Sodium Citrate; 1:1) were added drop by drop and incubated at 37°C for 15 min. Ten drops of fresh cold fixative methanol (Merck, Germany) and acetic acid (Merck, Germany) with a ratio of 3:1 were added while gently mixing. The tube was placed on ice for 15 min and centrifuged at 1000 rpm for 5 min. The supernatant was discarded and the pellet was re-washed with 5 ml fixative. After that, the supernatant was decanted and 200 μl of fixative was added. Next, a maximum of 3 drops of the suspended cells were dropped carefully from a height of approximately 0.5 meters onto the slide, and left at room temperature overnight to dry. The slides were stained with Giemsa (GIBCO, USA) working solution for 3–5 min and rinsed with distilled water. The slides were air-dried and then analysed.

### Statistical analysis

Analyses were performed using the Statistical Analysis System (SAS Institute 2003). The data were presented as mean ± standard deviation. It was analysed using the ANOVA procedure of SAS. Significant differences were further separated using Duncan’s multiple range test. Differences with a value of p<0.05 were considered to be statistically significant.

## Results

### Histopathology study

Histological examination of the breast tissues showed that ER, PR and HER2/neu status of tumoral tissue for MBC1 was ER^-^, PR^-^ and HER2^-^ (Triple negative), while for MBC2 was ER^-^, PR^-^ and HER2^+^ (Data not shown).

### Morphology and culture of primary breast cell lines

**Figure 1:**
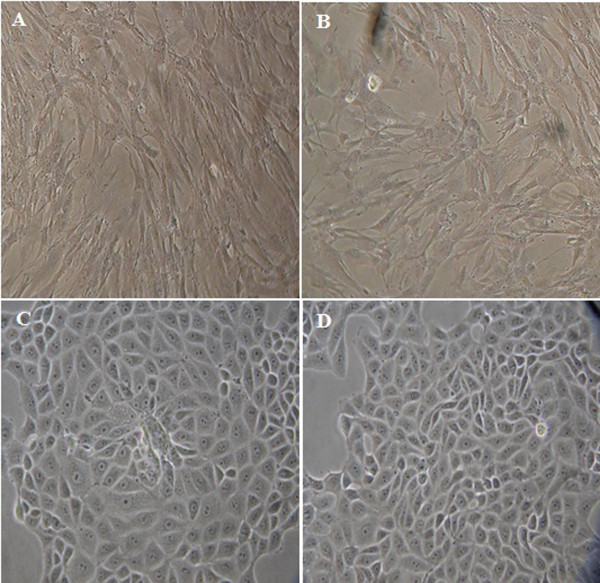
**Morphology of the MBC1 and MBC2 Cell Lines.** Panels (**A**) and (**B**) represent breast cancer MBC1-PN-2 and MBC2-PN-2, respectively; panels (**C**) and (**D**) represent breast cancer MBC1-PN-125 and MBC2-PN-85, respectively. Magnification, 40X; PN: passage number.

The population doubling time (PDT) of the first passage for the MBC1 and MBC2 was approximately 30 days after the initiation of culture. The morphology (Figure 1) of the two established human breast cell lines and the purity of the epithelial cells (data not shown) of the MBC1 and MBC2 human breast cell lines were determined. The growth medium for both MBC1 and MBC2 consisted of DMEM supplemented with 10% FBS and kept in a humidified incubator with 5% CO_2_ at 37°C. Cells were maintained in culture until passages 150, and then kept in liquid nitrogen. None of these established cell lines exhibited any signs of senescence and they were therefore considered immortalized. No sign of contamination, such as mycoplasma, fungi, or bacteria was observed.

### Clonogenic assay

**Figure 2:**
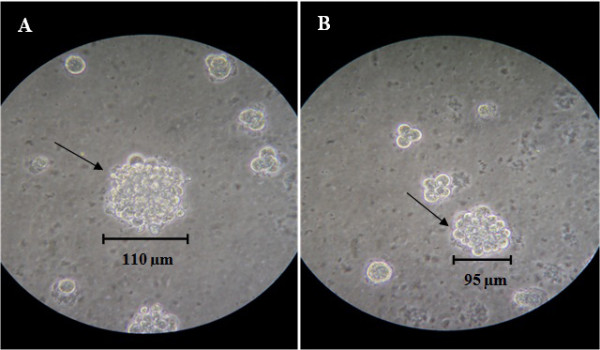
**Soft-agar Colony Formation Assay (Clonogenic Assay) of the MBC1 and MBC2 Cell Lines at 14 Days after Seeding.** Panels (**A**) and (**B**) represent MBC1 and MBC2 cell lines, respectively. Magnification, 10X.

**Figure 3:**
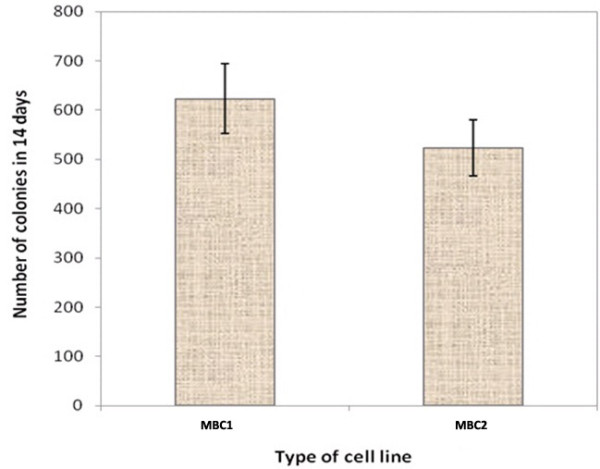
**Comparison of Colony Formation Rates of the MBC1 and MBC2 Cell Lines.** Cell colony numbers were determined in triplicates where the both cells showed no significant differences (p>0.05). Error bars represent the mean ± SD.

The MBC1 and MBC2 cell lines were able to produce colonies on the plate with similar cloning efficiencies (CE) of 1.074% and 1.048%, respectively (Figure 2). Both MBC1 and MBC2 cell lines produced a colony size of approximately 95–110 μm in diameter after 14 days on the soft agar. The cells also showed no significant difference (p>0.05) in the colony formation rate (Figure 3).

### Wound healing assay (Migration assay)

**Figure 4:**
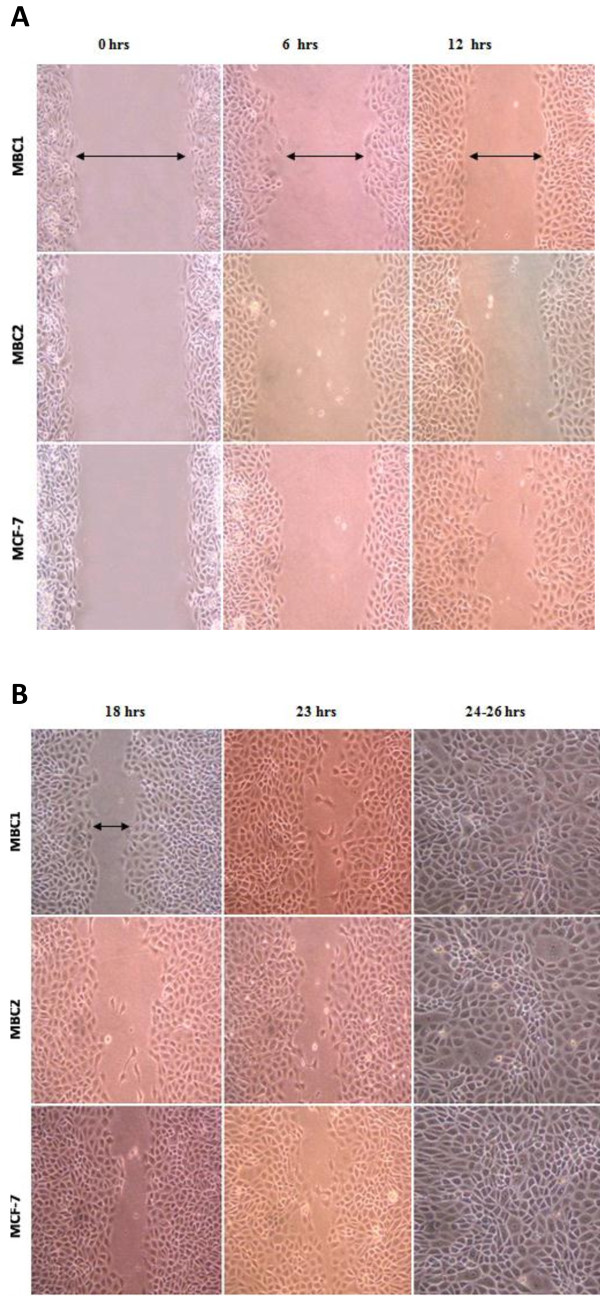
**A: Wound Healing Assay of the MBC1, MBC2 and MCF-7 Cell Lines at 0, 6 and 12 hrs of Culturing.** The result of growth rates in wound healing assays increased in the order of MBC2 < MBC1 < MCF-7, respectively. Magnification, 40X. **B**: Wound Healing Assay of the MBC1 and MBC2 Cell Lines at 18, 23 and 24–26 hrs of Culture. The result of growth rates in wound healing assays increased in the order of MBC2 < MBC1 < MCF-7, respectively. Magnification, 40X.

The migration of MBC1 and MBC2 cell lines across the wounds was calculated as a percentage of wound closure. The growth rates of wound-healing assays for MCF-7 and MBC2 showed the highest and lowest growth rates *in vitro* wound-healing assays, respectively (Figure 4A, B).

### Matrigel invasion assay

**Figure 5:**
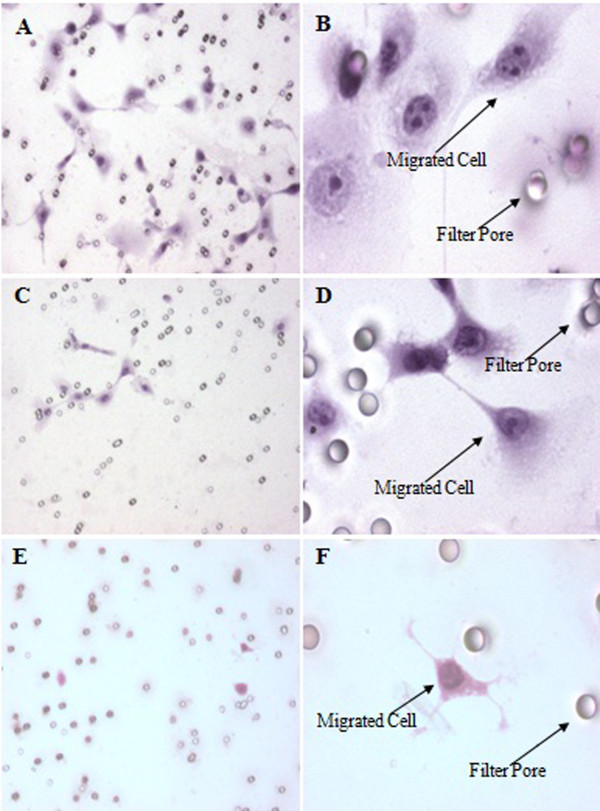
**Matrigel Invasion Assay of MBC1 and MBC2 Cell Lines as Compared to MCF-7.** Panels (**A**) and (**B**) show the invasion activity of MBC1, and panels (**C**) and (**D**) indicate the invasion activity of MBC2. Panels (**E**) and (**F**) demonstrate the invasion activity of MCF-7 as positive control. Magnification of panels **A**, **C **&**E** is 10X; **B**, **D** &**F** is 40X.

**Figure 6:**
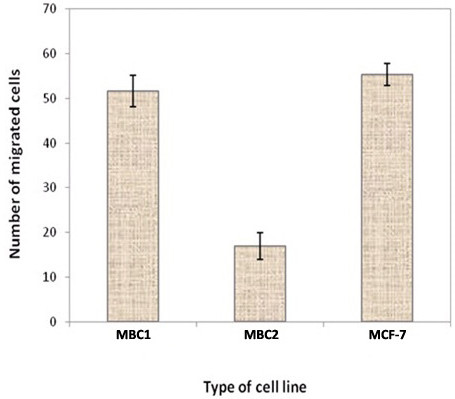
**Invasion Rates of the MBC1 and MBC2 Cell Lines as Compared with MCF-7.** Error bars indicate mean ± SD.

The invasion percentage of MBC1, MBC2 and MCF-7 were 0.110%, 0.040% and 0.116%, respectively (Figure 5, A-F). No significant differences were observed between MBC1 and MCF-7 (p>0.05), indicating the similar invasion ability of the MBC1 and MCF-7 cell lines to migrate through and adhere to the lower side of the filter *in vitro* condition. However, MBC2 demonstrated lower invasion activity compared to the MBC1 and MCF-7 (p<0.05) (Figure 6).

### Population doubling time (PDT)

**Figure 7:**
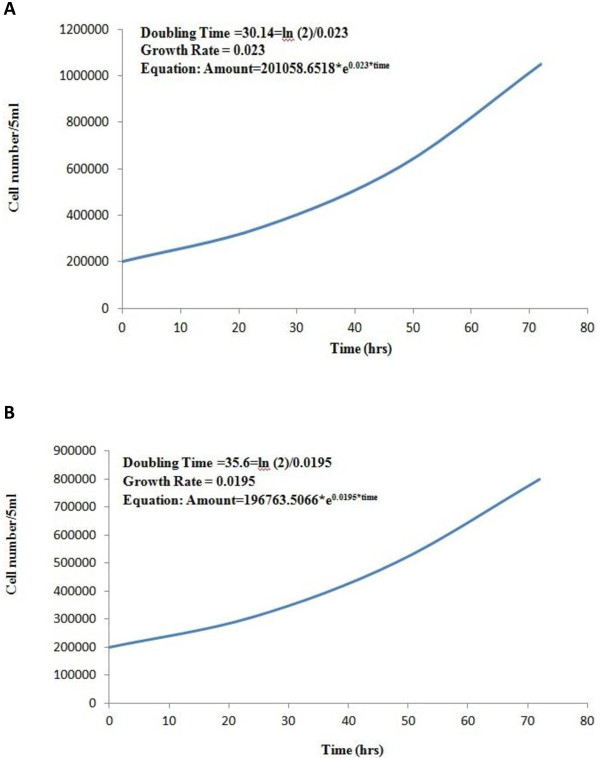
**A: Population Doubling Time (PDT) of the MBC1 Cell Lines.** Proliferation of the MBC1 was analysed by counting the total cell number at 0, 24, 48 and 72 time points. MBC1 proliferates *in vitro* with a PDT of 30.14 hours. **B**: Population Doubling Time (PDT) of the MBC2 Cell Lines. Proliferation of the MBC2 was analysed by counting the total cell number at 0, 24, 48 and 72 time points. MBC2 proliferates *in vitro* with a PDT of 35.6 hours.

The growth kinetics and the corresponding doubling time at 0, 24, 48 and 72 hrs of incubation for the MBC1 and MBC2 cell lines were determined at passage 30. The PDT of the MBC1 and MBC2 were 30.14 hrs and 35.6 hrs. The growth rate coefficients for the MBC1 and MBC2, representing the number of doublings per unit of time, were 0.023 (2.3%) and 0.0195 (1.95%), respectively (Figures 7A, B).

### DNA-profiling and amelogenin

**Figure 8:**
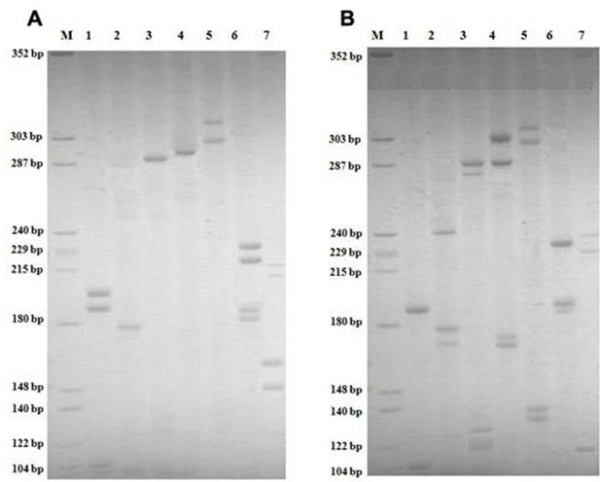
**Polyacrylamide Gel Electrophoresis of 13 CODIS STR Loci and Amelogenin for the MBC1 (Panel A) and MBC2 (Panel B) Cell Lines. (Panel A):** M: DNA molecular size marker. Lanes 1:[Amelogenin (106 bp), FGA (185 bp, 193 bp)], 2:[D8S1179 (165 bp)], 3:[D16S539 (290 bp)], 4:[D18S51 (292 bp)], 5:[CSF1PO (304 bp, 324 bp)], 6:[TH01 (181 bp, 185 bp), TPOX (225 bp, 236 bp)], and 7:[VWA (145 bp, 150 bp), D21S11 (213 bp, 218 bp)]. **(Panel B):** M: DNA molecular size marker. Lanes 1:[Amelogenin(106 bp), FGA(190 bp)], 2:[D8S1179 (171 bp, 179 bp), D7S820 (239 bp)], 3:[D3S1358 (122 bp, 124 bp), D16S539 (284 bp, 291 bp)], 4:[D13S317 (169 bp, 173 bp), D18S51(289 bp, 304 bp)], 5:[D5S818 (133 bp, 139 bp), CSF1PO (302 bp, 310 bp)], 6:[TH01 (189 bp, 193 bp), TPOX (232 bp], and 7:[VWA (122 bp), D21S11 (228 bp, 240 bp)].

DNA profiling using STR markers and Amelogenin of the cells were visualized on 10% polyacrylamide gels using silver staining method (Figure 8). The PCR product sizes were determined using Labworks software (UVP). No amplification was observed for the *D3S1358, D5S818* and *D13S317* loci in the MBC1 cells on polyacrylamide gel electrophoresis (PAGE), while all loci were amplified in the MBC2 cell line (Table 1).

**Table 1:** Allele assignment analysis of the amplified STR Loci and Amelogenin for the MBC1 and MBC2 Cell Lines

**STR locus**	**MBC1**	**MBC2**
*CSF1PO*	10, 15	9, 11
*FGA*	19, 21	20
*TH01*	4, 5	6, 7
*TPOX*	6, 9	8
*VWA*	15, 17	10
*D3S1358*	*-*^†^	14, 17
*D5S818*	*-*^†^	10, 11
*D7S820*	13	11
*D8S1179*	9	10, 12
*D13S317*	*-*^†^	8, 9
*D16S539*	*12*	*9, 11*
*D18S51*	*14*	*13, 17*
*D21S11*	26, 27	30, 33
*AMEL*	X, X	X, X

^†^ No STR loci identified for the MBC1 cells.

### Western blotting

**Figure 9:**
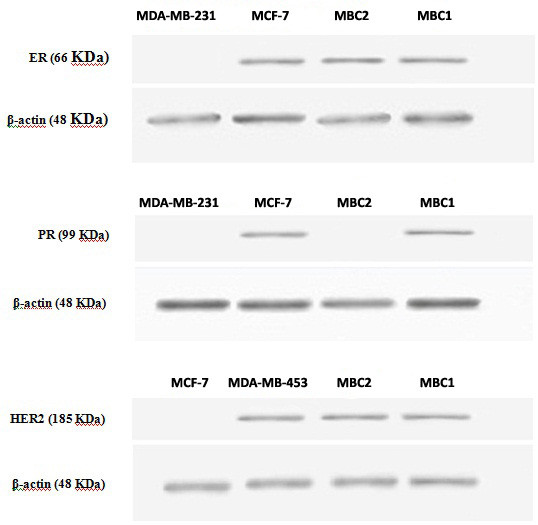
Detection of ER, PR, HER2 and β-actin expression in various breast cancer cell lines by western blotting.

ER expression was detected in the MBC1 and MBC2 cells and MCF-7 (as positive control). No band was observed for the MDA-MB-231(as negative control). Immunoblotting indicated the expression of PR for the MBC1 cells, but not expressed for the MBC2, while both MBC1 and MBC2 were positive for the expression of HER2. All cell lines expressed clear and distinct bands of β-actin, indicating the integrity of the assay (Figure 9).

### Fluorescence immunocytochemistry

**Figure 10:**
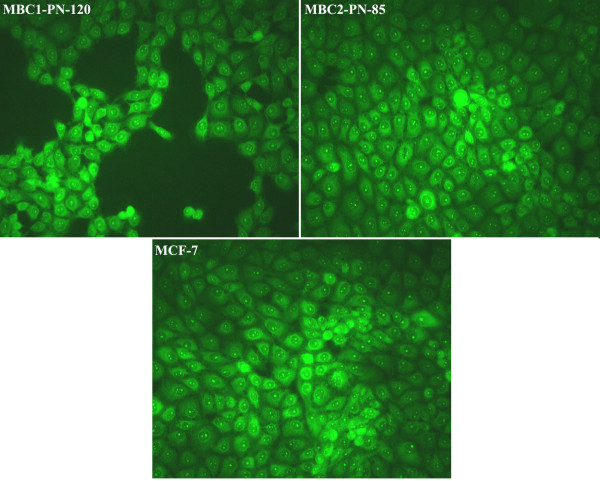
**Expression of ER in MBC1 and MBC2 Cell Lines, with MCF-7 as a Positive Control.** The expression of ER was shown in the MBC1-PN-120, MBC2-PN-85 and MCF-7 (as a positive control) with a localised nuclear staining pattern. Magnification, 20X.

**Figure 11:**
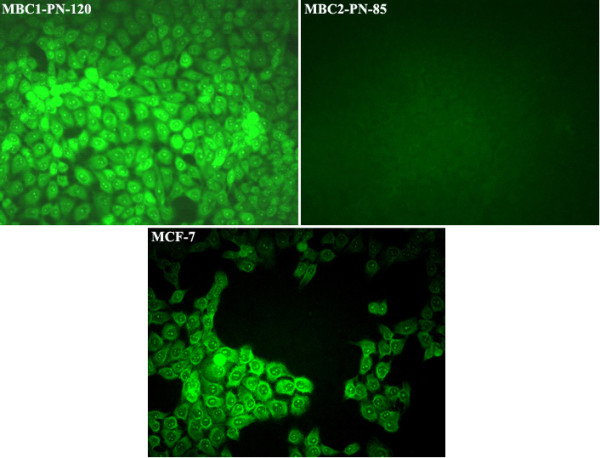
**Expression of PR in MBC1 and MBC2 Cell Lines, with MCF-7 as a Positive Control.** ICC/IF showed the expression of PR receptor with a localised nuclear staining pattern in the MBC1-PN-120 and MCF-7(as a positive control) but not in MBC2-PN-85. Magnification, 20X.

**Figure 12:**
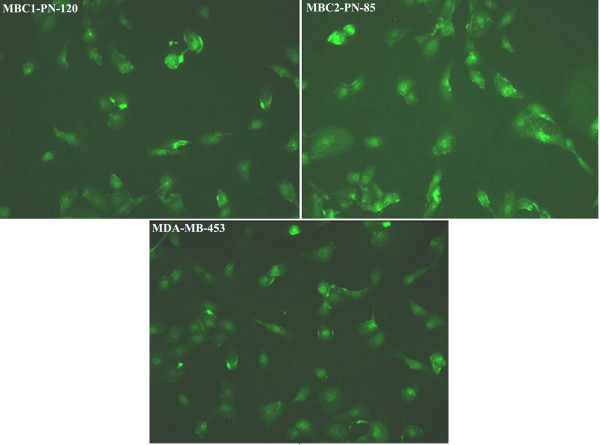
**Expression of HER2 in MBC1 and MBC2 Cell Lines, with MDA-MB-453 as a Positive Control.** The expression of HER2 with a localised staining pattern in the cell membranes was shown in the MBC1-PN-120, MBC2-PN-85 and MDA-MB-453(as a positive control). Magnification, 20X.

Fluorescence ICC shows the presence of ER in the MBC1, MBC2 and MCF-7 as localised nuclear staining pattern. ICC/IF analysis also revealed the expression of progesterone receptors (PR) in the MBC1 and MCF-7 cells with the same localised pattern. However, the MBC2 cell line was shown to be PR negative compared to MCF-7. The expression of HER2 receptor was further observed by fluorescence ICC in the cell membrane of the MBC1, MBC2 and MDA-MB-453 cell lines (Figures 10, 11, 12).

### Cell cycle analysis

**Figure 13:**
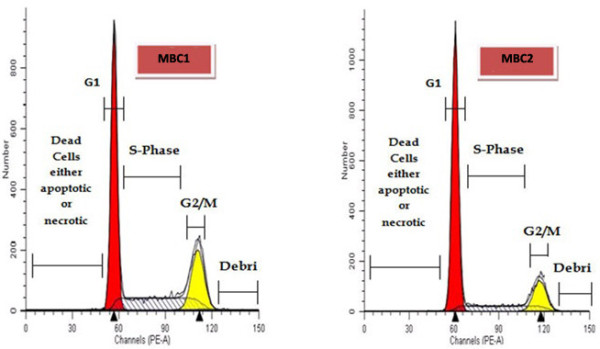
**Cell Cycle Analysis of the MBC1 and MBC2 Cell Lines by Flow Cytometry Histogram.** Enhancement in the S-phase fraction (SPF) demonstrates an unusual activity level of MBC1 proliferation compared to MBC2.

The number of the MBC1 cells showed an increase in the S-phase on the FCM histogram. The proliferation indices (P.I.) of the MBC1 cell line (P.I. 0.61) was approximately two times higher than the MBC2 (P.I. 0.32), indicating a more proliferative phenotype in the MBC1 cell line than MBC2 (Figure 13, Table 2).

**Table 2:** Comparison of DNA ploidy and cell cycle measurements of the MBC1 and MBC2 cell lines

	**MBC1**		**MBC2**	
	**Diploid**	**Tetraploid**	**Diploid**	**Tetraploid**
	**(83.68%)**	**(16.32%)**	**(92.68%)**	**(7.32%)**
**G0/G1**	61.88%	%100	75.24%	100%
**S**	30.12%	%0	16.76%	0%
**G2/M**	8%	%0	8%	0%
**Proliferation Index (P.I.)**	0.61		0.32	

### Cytogenetic analysis

**Figure 14:**
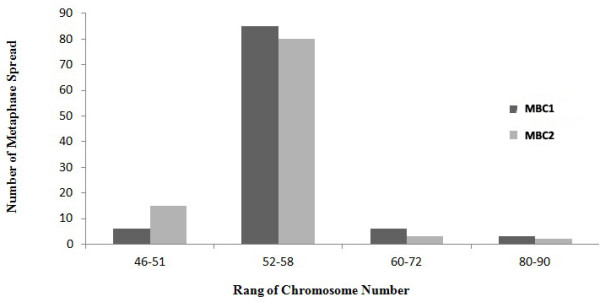
**Chromosomal Distribution Pattern of the MBC1 and MBC2 Cell Lines in 100 Metaphase Spreads.** A high percentage of the metaphase spreads showed chromosome numbers ranging from 52–58 in both MBC1 and MBC2 cell lines.

The G-banding karyotype of the metaphase spread was carried out. Karyotype analysis of the cells with the different cell passages revealed structural and numerical chromosomal abnormalities with chromosome numbers of 46 to 90, in which the majorities show a range of chromosomes between 52 to 58 including several unidentified chromosomes (Figure 14).

## Discussion

Both MBC1 and MBC2 are the first breast cancer cell lines established from Malaysian primary breast cancer tissues through spontaneous immortalization. The established cell lines grow as monolayers, adherent with consistent morphologically during subsequent cultures up to 150 passages, without any changes in the epithelial morphology such as MCF-7 and MDA-MB-231. According to data on other human primary cells, the senescence period of cells undergoing cycle arrest varies from 1 to 8 weeks [11]. However, both MBC1 and MBC2 cell lines bypassed this critical phase spontaneously after 4–5 weeks.

The MBC1 and MBC2 cell lines were able to grow in a defined DMEM medium [12] without any extra essential components, elements, or hormones. This is in contrast to for example, MCF-10A cell line requiring cholera toxin; MCF-7 and T-47D needing bovine insulin; and MDA-MB-435S needing ATCC-formulated Leibovitz’s L-15 Medium with bovine insulin and glutathione for growth [7,9,13,14].

The more established breast cancer cell lines such as MDA-MB-231 and MCF-7 were derived from malignant effusions [15] which may genetically differ from the original tumour [16,17]. However, the breast cancer cells, MBC1 and MBC2, were derived from primary infiltrating ductal breast tumours, the most frequently observed histological type [18]. Cells derived from primary breast tumours have a lower chance of establishment compared to cell lines derived from metastatic sites or pleural effusion [13].

The PDT of the MBC1 cells (30.14 hrs) is comparable with the established Italian BRC-230 breast cancer cell line [19] and French human breast cell line VHB-1 [20] with the PDT of 30.5 and 30 hrs, respectively; while the PDT of MBC2 (35.6 hrs) is similar to the Chinese BC-019, BC-020 breast cancer cell lines with the PDT of 36 hrs and 35 hrs, respectively [21]. The PDT of MBC1 and MBC2 is higher than the Italian breast cancer cell lines MAST, MA2 and MA3 with a PDT of 68, 70 and 78 hrs [22]; the human breast cancer cell lines UACC-812 and UACC-893 [23] with a doubling time of 100 hrs; the cell lines ZR-75-1, ZR-75-27, and ZR-75-30 from the malignant effusions of breast cancer with PDT of 80 hrs, 144 hrs and 110 hrs, respectively [24]. According to the results obtained by other researchers, one of the main reasons for the different PDTs of cell lines is the differences in cellular content rather than differences in the rates of proliferation [25]. The enhancement of the population doubling *in vitro* in the MBC1 and MBC2 human breast cell lines may be due to the existence and activity of tumourigenic stem cells [26] or most likely arises from the selection of faster-growing subclones from an initially heterogeneous population through frequent and incomplete trypsinisation of the cultures [27].

Both MBC1 and MBC2 cell lines produced a colony size of approximately 95–110 μm in diameter after day 14 on soft agar. Depending on the cell line and incubation time, different colony sizes have been reported. For instances, MCF-7 cell line forms 75–100 μm colonies after 11 days of growth and 100–200 μm in diameter by 21 days; T-47D cells form well-defined colonies with diameters of 80 μm by day 14 and 120 μm by day 21; MaTu cells grow well in soft agar with colony sizes of 50–75 μm diameters and sometimes greater than 100 μm diameters; MT-1 cells grow rapidly into colonies typically 70 μm in diameter by 11 days. However, some cell lines such as MT-3 and HS578T do not grow well in soft agar and therefore form only small colonies in the plate while some others like MC4000 cells produced no colony growth in soft agar [25].

Wound healing assay showed lower growth rate of the MBC2 cells compared to the MBC1 and MCF-7 as a control. The MBC1 cell line had similar growth rates with the control cell line MCF-7 in wound healing assay.

The results of entire 13 CODIS core STR loci were compared with the well-characterised and validated references provided by ATCC and JCRB, and those previously reported [10]. The two established cell lines have neither homology to each other nor with other STR-profiled breast cell lines, demonstrating the lack of any cross-contamination of cell lines with other studied cell lines such as MCF-7 [7], MDA-MB-231 [28], HeLa, HepG2, KB and MRC-5 [10]. DNA profiling using STR markers also confirmed the gender of tumour origin of MBC1 and MBC2 cell lines.

Expression studies revealed remarkable expression of HER2 protein in both established cell lines. The MBC1 cell line was also shown to be ER^+^/PR^+^ while the MBC2 was ER^+^/PR^-^ based on mRNA expression profiling. However, the tumour origins were shown ER^-^/PR^-^/HER2^-^ and ER^-^/PR^-^/HER2^+^ for the MBC1 and MBC2 cells, respectively. This may be due to a gain in secondary properties after culturing or that the MBC1 and MBC2 cells were derived from a small minority of ER^+^/PR^+^/HER2^+^ and ER^+^/PR^-^/HER2^+^ cells, respectively. The mechanism of this gain or loss during carcinogenesis is largely unknown but a very feasible hypothesis suggests a possible relationship between such deregulation and specific genetic changes [29].

Hence, these changes could be attributed to the Warburg effect, defined as an increased dependence on glycolysis for ATP synthesis, even in the presence of abundant oxygen, instead of a cell using the more effective pathway of OXPHOS [30]. The Warburg effect has been consistently observed in a wide spectrum of human cancers, and has been directly linked to the activation of oncogenes, and loss of tumor suppressor genes, which result in the deregulated conversion of glucose to lactate. Among the possible mechanisms, mitochondrial malfunction and hypoxia in the tumor microenvironment are considered two major factors contributing to the Warburg effect [31,32].

The MBC1 and MBC2 cells showed an SPF of 30.12% and 16.76% with proliferation index (PI) of 0.61 and 0.32, respectively, indicating a proportion of MBC2 cells were arrested in the G0/G1 phase, delaying the progression of the cell cycle and inhibiting cell proliferation compared to the MBC1.

The karyology study of the MBC1 and MBC2 cells demonstrated hyperdiploidy and complex rearrangement of their chromosomes. The fractured chromosomes were observed in different numbers in the MBC1 and MBC2. The hyperdiploidy (> 50 chromosomes) may arise from the fracture of the chromosomes. Cytogenetic analysis of MBC1 and MBC2 revealed an extensively rearranged hyperdiploid karyotype with unidentified chromosomes and a modal chromosome number of 52–58.

## Conclusions

Receptor changes may be due to loss or gain in secondary properties, activation of cancer stem cells and unknown specific genetic changes in tumoral cells. Regardless of receptor status, we hypothesize that, among heterogenous tumoral cells, only cells with low energy metabolism might survive and adjust easily to *in vitro* conditions. This is to date the first isolation and establishment of immortalised Malaysian primary breast cancer cell lines. Breast cancer cell lines which are currently used are mostly derived from the Caucasian population. The accessibility of two newly established human breast cell lines, MBC1 and MBC2, could provide us the important tools and new experimental models to study the gene expression patterns, efficacy of therapeutic drugs and prognostic and diagnostic markers, pathogenic mechanisms and biological behaviour of breast cancer *in vitro* and *in vivo*. These cell lines could also provide the groundwork to further investigate the activity of new anti-tumour agents in mono- or polychemotherapy *in vitro* or to elucidate the mechanisms of drug resistance in breast cancers in the future. They also would serve as prototype models of Malaysian breast carcinogenesis.

## Abbreviations

ER: Estrogen receptor; PR: Progesterone receptor; HER2/neu: Human epidermal growth factor receptor 2; PDT: Population doubling time; FBS: Fetal bovine serum; RT: Room temperature; CE: Cloning efficiency; SDS-PAGE: Sodium dodecyl sulfate polyacrylamide gel electrophoresis; ATCC: American type culture collection; P.I: Proliferation index; SPF: S-phase fraction; DMEM: Dulbecco’s modified eagle medium; PBS: Phosphate buffered saline; STR: Short tandem repeat; CODIS: Combined DNA index system; ICC: Immunocytochemistry.

## Competing interests

The authors declare that they have no competing interests.

## Authors’ contributions

BK carried out the experimental procedures and wrote the manuscript. RR conceived the project and supervised the study. FK and SA participated in the karyotyping and DNA profiling studies, respectively. NJ performed the statistical analysis. MH edited and revised the manuscript. SNA coordinated the collection of two invasive ductal breast carcinoma tissues of the patients and histopathological study. All authors read and approved the final manuscript.

## References

[B1] Zimonjic D Brooks MW Popescu N Weinberg RA Hahn WC Derivation of human tumor cells in vitro without widespread genomic instability Cancer Res 2001 61 24 8838 11751406

[B2] Hisham A Yip C Overview of breast cancer in Malaysian women: a problem with late diagnosis Asian J Surg 2004 27 2 130 133 10.1016/S1015-9584(09)60326-2 15140665

[B3] Hisham A Yip C Spectrum of breast cancer in Malaysian women: overview World journal of surgery 2003 27 8 921 923 10.1007/s00268-003-6976-x 12784146

[B4] Correa C Bertollo C Goes A Establishment and characterization of macl-1 and mgso-3 cell lines derived from human primary breast cancer Oncology Research Featuring Preclinical and Clinical Cancer Therapeutics 2009 17 10 473 482 10.3727/096504009789735404 19725227

[B5] Burdall S Hanby A Lansdown M Speirs V Breast cancer cell lines: friend or foe? Breast Cancer Research 2003 5 2 89 89 10.1186/bcr577 12631387 PMC154155

[B6] Osborne C Hobbs K Trent J Biological differences among MCF-7 human breast cancer cell lines from different laboratories Breast Cancer Res Treat 1987 9 2 111 121 10.1007/BF01807363 3620713

[B7] Soule H Vazguez J Long A Albert S Brennan M A human cell line from a pleural effusion derived from a breast carcinoma J Natl Cancer Inst 1973 51 5 1409 4357757 10.1093/jnci/51.5.1409

[B8] Engel LW Young NA Human breast carcinoma cells in continuous culture: a review Cancer Res 1978 38 11 Part 2 4327 212193

[B9] Keydar I Chen L Karby S Weiss F Delarea J Radu M Chaitcik S Brenner H Establishment and characterization of a cell line of human breast carcinoma origin Eur J Cancer 1979 15 5 659 670 10.1016/0014-2964(79)90139-7 228940

[B10] Azari S Ahmadi N Tehrani M Shokri F Profiling and authentication of human cell lines using short tandem repeat (STR) loci: Report from the National Cell Bank of Iran Biologicals 2007 35 3 195 202 10.1016/j.biologicals.2006.10.001 17254797

[B11] Lloyd A Limits to lifespan Nature Cell Biology 2002 4 2 E25 E27 10.1038/ncb0202-e25 11835050

[B12] Dulbecco R Freeman G Plaque production by the polyoma virus Virology 1959 8 3 396 10.1016/0042-6822(59)90043-1 13669362

[B13] Cailleau R Olivé M Cruciger Q Long-term human breast carcinoma cell lines of metastatic origin: preliminary characterization In Vitro Cellular & Developmental Biology-Plant 1978 14 11 911 915 10.1007/BF02616120 730202

[B14] Band V Zajchowski D Swisshelm K Trask D Kulesa V Cohen C Connolly J Sager R Tumor progression in four mammary epithelial cell lines derived from the same patient Cancer Res 1990 50 22 7351 1977518

[B15] Minafra S Morello V Glorioso F La Fiura A Tomasino R Feo S McIntosh D Woolley D A new cell line (8701-BC) from primary ductal infiltrating carcinoma of human breast Br J Cancer 1989 60 2 185 10.1038/bjc.1989.248 2548558 PMC2247044

[B16] Fogh J Human tumor lines for cancer research Cancer Investigation 1986 4 2 157 184 10.3109/07357908609038260 3518877

[B17] Pettengill O Lewko W Cell culture of human metastatic breast carcinomas: a review Molecular Biotherapy 1989 1 3 122 2690868

[B18] Vijver M Molecular genetic changes in human breast cancer Adv Cancer Res 1993 61 25 56 8346719 10.1016/s0065-230x(08)60954-9

[B19] Amadori D Bertoni L Flamigni A Savini S Giovanni C Casanova S Paola F Amadori A Giulotto E Zoli W Establishment and characterization of a new cell line from primary human breast carcinoma Breast Cancer Res Treat 1993 28 3 251 260 10.1007/BF00666586 8018954

[B20] Vandewalle B D’Hooghe M Savary J Vilain M Peyrat J Deminatti M Delobelle-Deroide A Lefebvre J Establishment and characterization of a new cell line (VHB-1) derived from a primary breast carcinoma J Cancer Res Clin Oncol 1987 113 6 550 558 10.1007/BF00390864 2824520 PMC12248367

[B21] Shen C Gu M Liang D Miao L Hu L Zheng C Chen J Establishment and characterization of three new human breast cancer cell lines derived from Chinese breast cancer tissues Cancer Cell International 2009 9 1 2 10.1186/1475-2867-9-2 19121212 PMC2646685

[B22] Zoli W Roncuzzi L Flamigni A Gruppioni R Sensi A Zini N Amadori D Gasperi-Campani A A new cell line from human infiltrating ductal carcinoma of the breast: establishment and characterization J Cancer Res Clin Oncol 1996 122 4 237 242 10.1007/BF01209652 8601577 PMC12200457

[B23] Meltzer P Leibovitz A Dalton W Villar H Kute T Davis J Nagle R Trent J Establishment of two new cell lines derived from human breast carcinomas with HER-2/neu amplification Br J Cancer 1991 63 5 727 10.1038/bjc.1991.164 1674877 PMC1972383

[B24] Engel L Young N Tralka T Lippman M O’Brien S Joyce M Establishment and characterization of three new continuous cell lines derived from human breast carcinomas Cancer Res 1978 38 10 3352 688225

[B25] Hambly R Double J Thompson M Bibby M Establishment and characterisation of new cell lines from human breast tumours initially established as tumour xenografts in NMRI nude mice Breast Cancer Res Treat 1997 43 3 247 258 10.1023/A:1005756632293 9150904

[B26] Han J Crowe D Tumor initiating cancer stem cells from human breast cancer cell lines Int J Oncol 2009 34 5 1449 19360358

[B27] Reile H Birnböck H Bernhardt G Spruß T Schönenberger H Computerized determination of growth kinetic curves and doubling times from cells in microculture Anal Biochem 1990 187 2 262 267 10.1016/0003-2697(90)90454-H 2382827

[B28] Cailleau R Young R Olive M Reeves W Jr Breast tumor cell lines from pleural effusions J Natl Cancer Inst 1974 53 3 661 4412247 10.1093/jnci/53.3.661 PMC7364228

[B29] Ito I Yoshimoto M Iwase T Watanabe S Katagiri T Harada Y Kasumi F Yasuda S Mitomi T Emi M Association of genetic alterations on chromosome 17 and loss of hormone receptors in breast cancer Br J Cancer 1995 71 3 438 10.1038/bjc.1995.89 7880720 PMC2033649

[B30] Warburg O On respiratory impairment in cancer cells Science 1956 124 3215 269 270 13351639

[B31] Vander Heiden MG Cantley LC Thompson CB Understanding the Warburg effect: the metabolic requirements of cell proliferation Science’s STKE 2009 324 5930) 1029 10.1126/science.1160809 PMC2849637 19460998

[B32] Kroemer G Pouyssegur J Tumor cell metabolism: cancer’s Achilles’ heel Cancer Cell 2008 13 6 472 482 10.1016/j.ccr.2008.05.005 18538731

